# Output correction factors for small static fields in megavoltage photon beams for seven ionization chambers in two orientations — perpendicular and parallel

**DOI:** 10.1002/mp.13894

**Published:** 2019-11-25

**Authors:** Božidar Casar, Eduard Gershkevitsh, Ignasi Mendez, Slaven Jurković, M. Saiful Huq

**Affiliations:** ^1^ Department for Dosimetry and Quality of Radiological Procedures Institute of Oncology Ljubljana Ljubljana Slovenia; ^2^ Medical Physics Service North Estonia Medical Centre Tallinn Estonia; ^3^ Medical Physics Department University Hospital Rijeka Rijeka Croatia; ^4^ Department of Physics and Biophysics Faculty of Medicine University of Rijeka Rijeka Croatia; ^5^ Department of Radiation Oncology University of Pittsburgh School of Medicine and UPMC Hillman Cancer Center Pittsburgh PA USA

**Keywords:** ionization chamber, orientation, output correction factor, small field

## Abstract

**Purpose:**

The goal of the present work was to provide a large set of detector‐specific output correction factors for seven small volume ionization chambers on two linear accelerators in four megavoltage photon beams utilizing perpendicular and parallel orientation of ionization chambers in the beam for nominal field sizes ranging from 0.5 cm^2^ × 0.5 cm^2^ to 10 cm^2 ^× 10 cm^2^. The present study is the second part of an extensive research conducted by our group.

**Methods:**

Output correction factors $k_{Q_{\mathrm{clin}},Q_{\mathrm{ref}}}^{f_{\mathrm{clin}},f_{\mathrm{ref}}}$ were experimentally determined on two linacs, Elekta Versa HD and Varian TrueBeam for 6 and 10 MV beams with and without flattening filter for nine square fields ranging from 0.5 cm^2 ^× 0.5 cm^2 ^to 10 cm^2 ^× 10 cm^2^, for seven mini and micro ionization chambers, IBA CC04, IBA Razor, PTW 31016 3D PinPoint, PTW 31021 3D Semiflex, PTW 31022 3D PinPoint, PTW 31023 PinPoint, and SI Exradin A16. An Exradin W1 plastic scintillator and EBT3 radiochromic films were used as the reference detectors.

**Results:**

For all ionization chambers, values of output correction factors $k_{Q_{\mathrm{clin}},Q_{\mathrm{ref}}}^{f_{\mathrm{clin}},f_{\mathrm{ref}}}$ were lower for parallel orientation compared to those obtained in the perpendicular orientation. Five ionization chambers from our study set, IBA Razor, PTW 31016 3D PinPoint, PTW 31022 3D PinPoint, PTW 31023 PinPoint, and SI Exradin A16, fulfill the requirement recommended in the TRS‐483 Code of Practice, that is, $0.95<k_{Q_{\mathrm{clin}},Q_{\mathrm{ref}}}^{f_{\mathrm{clin}},f_{\mathrm{ref}}}<1.05$, down to the field size 0.8 cm^2 ^× 0.8 cm^2^, when they are positioned in parallel orientation; two of the ionization chambers, IBA Razor and PTW 31023 PinPoint, satisfy this condition down to the field size of 0.5 cm^2^ × 0.5 cm^2^.

**Conclusions:**

The present paper provides experimental results of detector‐specific output correction factors for seven small volume ionization chambers. Output correction factors were determined in 6 and 10 MV photon beams with and without flattening filter down to the square field size of 0.5 cm^2^ × 0.5 cm^2^ for two orientations of ionization chambers — perpendicular and parallel. Our main finding is that output correction factors are smaller if they are determined in a parallel orientation compared to those obtained in a perpendicular orientation for all ionization chambers regardless of the photon beam energy, filtration, or linear accelerator being used. Based on our findings, we recommend using ionization chambers in parallel orientation, to minimize corrections in the experimental determination of field output factors. Latter holds even for field sizes below 1.0 cm^2^ × 1.0 cm^2^, whenever necessary corrections remain within 5%, which was the case for several ionization chambers from our set.

TRS‐483 recommended perpendicular orientation of ionization chambers for the determination of field output factors. The present study presents results for both perpendicular and parallel orientation of ionization chambers. When validated by other researchers, the present results for parallel orientation can be considered as a complementary dataset to those given in TRS‐483.

## 1 Introduction

A new international Code of Practice (CoP) for reference and relative dosimetry in high energy small static photon fields, TRS‐483, has recently been published jointly by the International Atomic Energy Agency (IAEA) and the American Association of Physicists in Medicine (AAPM).^1^ A summary of this CoP has also been published by Palmans et al.^2^ The formalism recommend in the CoP is a slightly modified version of the formalism proposed by Alfonso et al.^3^ Field output factor $\Omega_{Q_{\mathrm{clin}},Q_{\mathrm{ref}}}^{f_{\mathrm{clin}},f_{\mathrm{ref}}}$ for a particular clinical field $f_{\mathrm{clin}}$ and reference field $f_{\mathrm{ref}}$ can be determined from the ratio of absorbed doses in both fields, and is given by

(1) $$\begin{array}{c} \Omega_{Q_{clin,}Q_{\mathrm{ref}}}^{f_{\mathrm{clin}},f_{\mathrm{ref}}}=\frac{D_{w,Q_{\mathrm{clin}}}^{f_{\mathrm{clin}}}}{D_{w,Q_{\mathrm{ref}}}^{f_{\mathrm{ref}}}} \end{array}$$

where $Q_{\mathrm{clin}}$ and $Q_{\mathrm{ref}}$ denote beam quality in the clinical and reference fields, respectively. The notation $f_{\mathrm{ref}}$ can refer to either a machine‐specific reference (msr) field or in the case of conventional linear accelerators where a 10 cm^2^ × 10 cm^2^ field can be set, the reference field size $f_{\mathrm{ref}}$ is equal to 10 cm^2^ × 10 cm^2^. In the present study, $f_{\mathrm{ref}}$=10 cm^2^ × 10 cm^2^.

For large clinical fields, a quotient of the absorbed doses in clinical and reference fields can be approximated by the ratio of detector readings $M_{Q_{\mathrm{clin}}}^{f_{\mathrm{clin}}}$ and $M_{Q_{\mathrm{ref}}}^{f_{\mathrm{ref}}}$ as

(2) $$\begin{array}{c} \frac{D_{w,Q_{\mathrm{clin}}}^{f_{\mathrm{clin}}}}{D_{w,Q_{\mathrm{ref}}}^{f_{\mathrm{ref}}}}\approx \frac{M_{Q_{\mathrm{clin}}}^{f_{\mathrm{clin}}}}{M_{Q_{\mathrm{ref}}}^{f_{\mathrm{ref}}}} \end{array}$$

However, such an approximation does not hold true in small fields, because in these cases, various field size‐dependent perturbation factors and volume averaging factors become significant. This requires that the ratio of charge readings in Eq. (2) is multiplied by a detector‐specific output correction factor $k_{Q_{\mathrm{clin}},Q_{\mathrm{ref}}}^{f_{\mathrm{clin}},f_{\mathrm{ref}}}$ for the determination of field output factors. Therefore, in the case of small fields, the correct expression for field output factors is given by Eq. (3)

(3) $$\begin{array}{c} \Omega_{Q_{\mathrm{clin}},Q_{\mathrm{ref}}}^{f_{\mathrm{clin}},f_{\mathrm{ref}}}=\frac{M_{Q_{\mathrm{clin}}}^{f_{\mathrm{clin}}}}{M_{Q_{\mathrm{ref}}}^{f_{\mathrm{ref}}}}k_{Q_{\mathrm{clin}},Q_{\mathrm{ref}}}^{f_{\mathrm{clin}},f_{\mathrm{ref}}} \end{array}$$

Combining Eqs. (1) and (3), we get a general equation for detector‐specific output correction factors derived from a ratio of true doses and detector readings in clinical and reference field as

(4) $$\begin{array}{c} k_{Q_{clin,}Q_{\mathrm{ref}}}^{f_{clin,}f_{\mathrm{ref}}}=\frac{D_{w,Q_{\mathrm{clin}}}^{f_{\mathrm{clin}}}/D_{w,Q_{\mathrm{ref}}}^{f_{\mathrm{ref}}}}{M_{Q_{\mathrm{clin}}}^{f_{\mathrm{clin}}}/M_{Q_{\mathrm{ref}}}^{f_{\mathrm{ref}}}} \end{array}$$

In addition to the new formalism for dosimetry in small static photon fields, TRS‐483 provided a large set of data for detector‐specific output correction factors for solid‐state detectors and cylindrical ionization chambers determined in 6 and 10 MV photon beams from various types of linear accelerators.^1^

**Figure 1:**
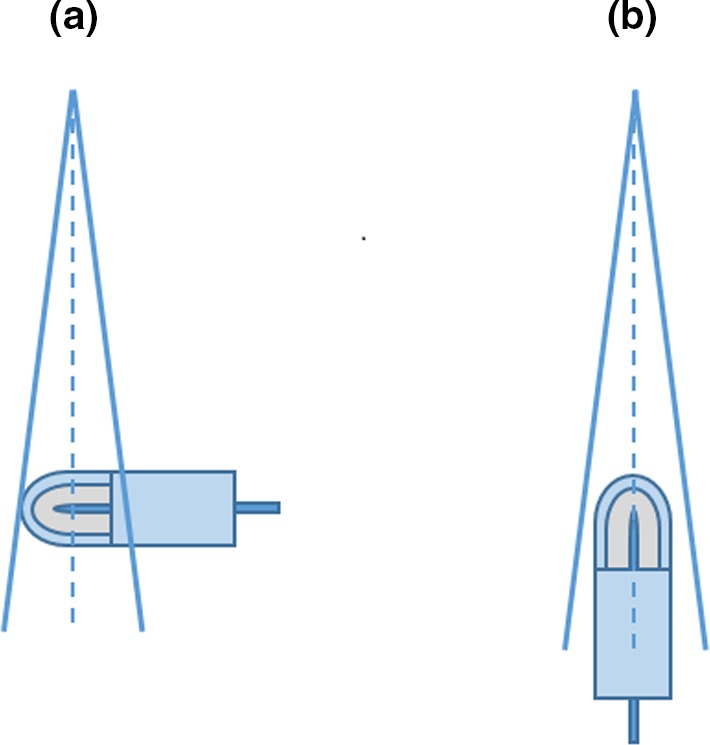
Schematic drawing of two alternative orientations of ionization chamber for measurement of field output factors and determination of output correction factors: (a) perpendicular orientation as described in the text and recommended in the TRS‐483 CoP and (b) parallel orientation, which is not recommended in the TRS‐483 CoP because of lack of output correction factor data for ionization chambers placed in this orientation. [Color figure can be viewed at http://www.wileyonlinelibrary.com]

TRS‐483 recommends that the determination of field output factors using cylindrical ionization chambers should be made from measurements made in the perpendicular orientation, that is, the longest axis of the chamber should be oriented perpendicular to the beam central axis as shown in Fig. 1(a) (perpendicular orientation). The reasons for recommending perpendicular orientation for the determination of field output factors in small fields is that there was a lack of high quality data in the published literature for output correction factors determined in parallel orientation for various combinations of chambers and beam energies obtained from different linear accelerators.^4^ Indeed, while the determination of output correction factors for ionization chambers in the perpendicular orientation has been extensively investigated by a number of research groups and for a range of ionization chambers,^5^, ^6^, ^7^, ^8^, ^9^, ^10^, ^11^, ^12^, ^13^, ^14^, ^15^, ^16^, ^17^, ^18^, ^19^, ^20^, ^21^, ^22^, ^23^, ^24^, ^25^, ^26^, ^27^, ^28^, ^29^, ^30^ only a few studies have reported results for $k_{Q_{\mathrm{clin}},Q_{\mathrm{ref}}}^{f_{\mathrm{clin}},f_{\mathrm{ref}}}$ for a few small volume ionization chambers^14^, ^15^, ^16^, ^18^, ^19^, ^31^ in small fields determined in parallel or for both orientations.

The orientation of the ionization chamber may not be crucial for the accuracy of relative measurements in large fields where lateral charged particle equilibrium (LCPE) exists on the beam central axis. However, in small fields below a size of about 2.0 cm, the selection of ionization chambers and choice of orientation of the longest axis of the chamber with respect to the beam central axis is important for performing field output factor measurements because of the onset of lack of LCPE, where the dimensions of ionization chambers, even small ones, become comparable to the field sizes.

Many air‐filled ionization chambers are designed such that they have larger cavity lengths along the direction of the chamber’s long axis compared to the cavity dimension in the radial direction. Depending on chamber design and orientation used for measurements of field output factors, the volume averaging effect can be significant if a chamber is placed with its stem perpendicular to the beam central axis. Furthermore, some ionization chambers show considerable stem effect, which can be minimized if the chamber is oriented with its stem parallel to the beam central axis, that is, in parallel orientation.^1^

The goal of the present work was twofold. First, we aimed to provide a large set of detector‐specific output correction factors for seven small volume ionization chambers/two linear accelerators/four photon beams combinations, utilizing perpendicular orientation as shown in Fig. 1(a). These results provide a valuable supplement to the TRS‐483 dataset, and serve as validation of dataset for three chambers included in the TRS‐483 Code of Practice.

Secondly, output correction factors for all seven small volume ionization chambers were also determined in the parallel orientation [Fig. 1(b)]. To the best of our knowledge, no similar comprehensive study for the determination of output correction factors in both orientation of ionization chambers in megavoltage photon beams has been published. The present set of data can be considered as a valuable contribution to the literature and can provide a basis for recommending parallel orientation of ionization chambers commonly used in clinic for measurement of field output factors in small static fields in megavoltage photon beams.

It should be noted that the present work is the second part of an extensive research conducted by our group. In the first part, we discussed the determination of field output factors and output correction factors for seven solid‐state detectors using two reference detectors.^32^ The same methodology from that part of the study was also followed in the present paper.

## 2 Materials and Methods

The experimental equipment and methodology used in the present study are the same as those used in the first part of our study, where we gave detailed discussion about the determination of field output factors using plastic scintillator Exradin W1 (Standard Imaging, Middleton, WI, USA) and radiochromic films EBT3 (Ashland Inc., Wayne, NJ, USA), as reference detectors and output correction factors for six diodes and a microdiamond detector.^32^, ^33^ Therefore, only a brief discussion on the salient features of the methodology will be given here.

Output correction factors for ionization chambers determined in this study represent the “total” correction factors for a particular detector and comprise individual correction factors for all contributing effects including volume averaging, polarity, and recombination.

### 2.1 Ionization chambers

Seven mini and micro ionization chambers were selected for the determination of detector‐specific output correction factors in small static fields in megavoltage photon beams: IBA CC04 and IBA Razor (IBA Dosimetry, Schwarzenbruck, Germany), PTW 31016 3D PinPoint, PTW 31021 3D Semiflex, PTW 31022 3D PinPoint, PTW 31023 PinPoint (PTW, Freiburg, Germany), and SI Exradin A16 (Standard Imaging, Middleton, WI, USA). The selection was based on their physical dimensions, characteristics, and suitability for clinical use, following the recommendations given in TRS‐483. Two ionization chambers included in the study, IBA Razor and SI Exradin A16, are classified as micro ionization chambers with active volume V ≤ 0.01 cm^3^, while the rest of the chambers belong to the group of small, sometimes named as mini ionization chambers having active volumes ranging from 0.01 cm^3^ < V < 0.3 cm^3^. Table 1 lists their physical properties and dimensions.

**Table 1:** Summary of physical properties of seven ionization chamber used in this study.

Detector type	Cavity volume (cm^3^)	Cavity length/radius (mm)	Wall material	Wall thickness (g/cm^2^)	Central electrode
IBA CC04	0.04	3.6/2.0	C552	0.070	C‐552
IBA Razor	0.01	3.6/1.0	C552	0.088	Graphite
PTW 31016 PinPoint 3D	0.016	2.9/1.45	PMMA + Graphite	0.085	Aluminium
PTW 31021 Semiflex 3D	0.07	4.8/2.4	PMMA + Graphite	0.084	Aluminium
PTW 31022 PinPoint 3D	0.016	2.9/1.45	PMMA + Graphite	0.084	Aluminium
PTW 31023 PinPoint	0.015	5.0/1.0	PMMA + Graphite	0.085	Aluminium
SI Exradin A16	0.007	2.4/1.2	C552	0.088	Steel^a^

a Silver plated and copper clad steel wire.

It should be noted that PTW 31022 3D PinPoint and PTW 31023 PinPoint chambers superseded previous models PTW 31016 3D PinPoint and PTW 31014 PinPoint, respectively. The newer models are geometrically and constructionally almost identical to their predecessors with the exception of the diameter of the central electrode which has been increased from 0.3 mm (old models) to 0.6 mm (new models).^34^, ^35^ There is also a difference in the nominal chamber bias voltage as specified by the manufacturer: for PTW 31023 PinPoint chamber, the nominal bias voltage is 200 V, while for the older model 31014 PinPoint, the nominal bias voltage is 400 V. Similarly, for PTW 31022 3D PinPoint chamber, the nominal voltage is 300 V, while for older model 31016, the nominal voltage is 400 V. Throughout the present study, the manufacturer’s specification regarding the nominal bias voltages was always followed.

Similarly, IBA Razor ionization chamber superseded previous model IBA CC01. However, in this case, there is significant constructional difference between both models: the new model has been designed with a graphite electrode while the old model is built with a steel electrode. The rest of the characteristics of the new model, that is, cavity dimensions, wall material, and wall thickness, remained the same as the old one.

### 2.2 Experimental setup

Dosimetry measurements for the determination of output correction factors were performed at two hospitals on two different linear accelerators, Elekta Versa HD^TM^ (Elekta AB, Stockholm, Sweden) equipped with Agility™ MLC system and Varian TrueBeam^TM^ (Varian Medical Systems, Palo Alto, CA, USA) equipped with Millenium™ MLC system, using photon beams of nominal energies 6 and 10 MV. Both MLC systems have leaves with 0.5 cm width in the central part of the radiation fields. Beams with flattening filters (WFF) as well as flattening filter free (FFF) beams were used for all measurements, and are denoted hereafter as 6 MV WFF, 6 MV FFF, 10 MV WFF, and 10 MV FFF. The measurement geometry consisted of an isocentric setup with a source‐to‐surface (SSD) distance of 90 cm, a depth of 10 cm, and gantry at 0°. For each point measurement, 100 MU was delivered to nine square fields with nominal side lengths of 0.5, 0.8, 1.0, 1.5, 2.0, 3.0, 4.0, 5.0, and 10.0 cm. On Elekta linac, radiation fields were shaped with MLC in cross‐line (x) direction and with jaws in the in‐line (y) direction. On Varian TrueBeam linac, radiation fields were collimated using the linac jaws in both axes, x and y. A conventional reference field *f_ref_* = 10 × 10 cm^2^ was used as the reference field for the calculation of detector‐specific output correction factors. At least three measurements of collected charge were taken for each setup, using a reference class PTW Unidos*^webline^* (PTW, Freiburg, Germany) electrometer. In the case of low signals, the range of the electrometer was adjusted appropriately. Measured raw signals were corrected for environmental conditions (temperature and pressure), whenever necessary. No other corrections (e.g., corrections for volume averaging, polarity, and recombination effect) were applied to the measured data obtained with ionization chambers.

A 3D water phantom (Blue Phantom 2, IBA Dosimetry, Schwarzenbruck, Germany) was used for the measurements on the Elekta Versa HD linac, while an MP3‐M water phantom (PTW, Freiburg, Germany) was used for measurements in the Varian TrueBeam linac.

Before measurements, each ionization chamber was positioned with its effective point of measurement at the reference depth of 10 cm. Two sets of measurements were performed: (a) with the chamber’s stem perpendicular to the beam axis as shown in Fig. 1(a) and (b) with its stem parallel to the beam axis as shown schematically in Fig. 1(b). For perpendicular orientation, the effective point of measurement in the vertical direction (along z axis) was taken as displaced by 0.6r (r is the internal radius of the chamber’s cavity) above the reference point of the chamber located on the chamber’s axis as specified by the manufacturer. For parallel orientation, effective point of measurement coincided with the reference point of the chamber specified by the manufacturer and measured from the tip of the chamber. Lateral alignment of ionization chambers along the beam central axis was done for each chamber separately in 3D water phantom following three steps listed below after the initial setup was done using room lasers:

1. CAX alignment, following the procedure within the integrated softwares Mephysto mc^2^ (for PTW MP3‐M water phantom) and myQA Accept (for IBA Blue Phantom 2);
2. repositioning (centering) of the ionization chamber after acquiring lateral beam profiles along cross‐line and in‐line directions;
3. finally, each detector was moved using manual mode in 0.2 mm (0.1 mm if necessary) steps along both, x and y axes, and irradiated every time with 100 MU to verify the position where the collected charge was maximal.

The position where the collected charge reached the highest value was assumed to lie at the peak of the beam profile along the beam central axis, that is, the center of the radiation field. Thus, obtained position of the ionization chamber was considered as the correct one and was used for the subsequent measurements of output factors. The above procedure for lateral alignment of detectors was done separately for each photon beam and for the two smallest fields (0.5 and 0.8 cm). Similar alignment procedure is also recommended in the ICRU Report 91.^36^ Lateral beam profiles along cross‐line and in‐line directions were acquired using the same orientation of the ionization chamber as it was used for subsequent point measurements. This means that for perpendicular orientation, we performed beam profile measurements in one of the directions (in‐line direction, which coincides with the principal chamber’s axis) which is not recommended in the TRS‐483 (see fig. 18 (3) in TRS‐483). To the contrary, for parallel orientation, the beam profiles in both directions were acquired using the guidance from the TRS‐483. For subsequent point measurements, we had to keep the same orientation as it was used during the scanning. This means that for the determination of field output factors for perpendicular orientation, the recommendations of TRS‐483 were followed. However, for measurements using parallel orientation, the recommendations of TRS‐483 were not followed.

As suggested by Cranmer‐Sargison et al.,^37^ and adopted by TRS‐483, the equivalent square small field size $S_{\mathrm{clin}}$ was calculated according to

(5) $$\begin{array}{c} S_{\mathrm{clin}}=\sqrt{A\times B} \end{array}$$

where *A* and *B* correspond to the radiation field width at half maximum (FWHM) in the in‐line and cross‐line directions, respectively, which were determined using EBT3 film measurements in the present work.^32^, ^33^ Full description of the methodology can be found in our previous work.^32^

### 2.3 Output correction factors

For every ionization chamber and each measured equivalent square small field size $S_{\mathrm{clin}}$, discrete values of output correction factors $k_{Q_{\mathrm{clin}},Q_{\mathrm{ref}}}^{f_{\mathrm{clin}},f_{\mathrm{ref}}}\left(S_{\mathrm{clin}}\right)$ were calculated using the following equation

(6) $$\begin{array}{c} k_{Q_{\mathrm{clin}},Q_{\mathrm{ref}}}^{f_{\mathrm{clin}},f_{\mathrm{ref}}}\left(S_{\mathrm{clin}}\right)=\frac{\Omega_{Q_{\mathrm{clin}},Q_{\mathrm{ref}}}^{f_{\mathrm{clin}},f_{\mathrm{ref}}}}{M_{Q_{\mathrm{clin}}}^{f_{\mathrm{clin}}}/M_{Q_{\mathrm{ref}}}^{f_{\mathrm{ref}}}} \end{array}$$

where $M_{Q_{\mathrm{clin}}}^{f_{\mathrm{clin}}}$ and $M_{Q_{\mathrm{ref}}}^{f_{\mathrm{ref}}}$ denote chamber readings in clinical and reference fields, respectively. Discrete values of field output factors $\Omega_{Q_{\mathrm{clin}},Q_{\mathrm{ref}}}^{f_{\mathrm{clin}},f_{\mathrm{ref}}}$ were obtained from the analytical function $\Omega \left(S_{\mathrm{clin}}\right)$ proposed by Sauer and Wilbert,^27^

(7) $$\begin{array}{c} \Omega \left(S_{\mathrm{clin}}\right)=P_{\infty }\frac{S_{\mathrm{clin}}^{n}}{l^{n}+S_{\mathrm{clin}}^{n}}+S_{\infty }\left(1-e^{-b\cdot S_{\mathrm{clin}}}\right) \end{array}$$

which was fitted to the measured data obtained with EBT3 films and W1 plastic scintillator. At least three measurements were taken with each detector (W1 plastic scintillator in the parallel orientation) assuming that both detectors are water equivalent and can be considered as reference detectors for the purpose of our study. While W1 plastic scintillator may exhibit minor deviations from perfect water equivalence for small fields,^38^ we did not consider that possibility as pertinent for our study. Film doses were calculated using the Multigaussian method implemented in Radiochromic.com v3.0 (Radiochromic SL, Benifaió, Spain).^33^ Detailed methodology for the determination of field output factors using two reference detectors, EBT3 films and W1 plastic scintillator, is provided in the paper by Casar et al.^32^

For the functional presentation of output correction factors, discrete values of $k_{Q_{\mathrm{clin}},Q_{\mathrm{ref}}}^{f_{\mathrm{clin}},f_{\mathrm{ref}}}\left(S_{\mathrm{clin}}\right)$ determined by Eq. (6) were fitted by the analytical function published in TRS‐483

(8) $$\begin{array}{c} k\left(S_{\mathrm{clin}}\right)=\frac{1-e^{-\frac{10-a}{b}}}{1-e^{-\frac{S_{\mathrm{clin}}-a}{b}}}+c\cdot \left(S_{\mathrm{clin}}-10\right) \end{array}$$

with fitting coefficients, a, b, c. Instead of symbol S, which is used in TRS‐483, symbol $S_{\mathrm{clin}}$ was used here instead, to emphasize that in the present study, equivalent square small field sizes were applied without exception. Subscripts and superscripts are omitted in the notations for output correction factors in Eq. (8) to indicate that in this case, output correction factors have functional form, unlike discrete values determined by the Eq. (6).

To investigate differences between output correction factors determined in perpendicular and in parallel orientations ($k_{\mathrm{perp}}$ and $k_{\mathrm{para}}$), ratios $k_{\mathrm{perp}}/k_{\mathrm{para}}$ were calculated using Eq. (6), resulting in

(9) $$\begin{array}{c} \frac{k_{\mathrm{perp}}}{k_{\mathrm{para}}}=\frac{{\left(M_{Q_{\mathrm{clin}}}^{f_{\mathrm{clin}}}/M_{Q_{\mathrm{ref}}}^{f_{\mathrm{ref}}}\right)}_{\mathrm{para}}}{{\left(M_{Q_{\mathrm{clin}}}^{f_{\mathrm{clin}}}/M_{Q_{\mathrm{ref}}}^{f_{\mathrm{ref}}}\right)}_{\mathrm{perp}}} \end{array}$$

where ${\left(M_{Q_{\mathrm{clin}}}^{f_{\mathrm{clin}}}/M_{Q_{\mathrm{ref}}}^{f_{\mathrm{ref}}}\right)}_{\mathrm{para}}$ and ${\left(M_{Q_{\mathrm{clin}}}^{f_{\mathrm{clin}}}/M_{Q_{\mathrm{ref}}}^{f_{\mathrm{ref}}}\right)}_{\mathrm{perp}}$ present measured ratios of readings in parallel and perpendicular orientation of ionization chambers, respectively.

### 2.4 Volume averaging considerations

The primary reason for the differences in output correction factors measured with different ionization chambers is the volume averaging effect of the different detectors. It is apparent that chambers with larger cavity volumes will have larger volume averaging correction factors $k_{\mathrm{vol}}$ compared to those with smaller volumes. However, some chambers with (almost) equal cavity volumes can have significantly different $k_{\mathrm{vol}}$, since their cavity dimensions in longitudinal and radial axes, $d_{L}$ and $d_{R}$, differ. Therefore, differences in $k_{\mathrm{vol}}$ will be reflected also in differences of total output correction factors.

The fact that output correction factors may depend significantly on the ratios $d_{L}/d_{R}$, ratios of $k_{\mathrm{perp}}/k_{\mathrm{para}}$ was analyzed in more detail for two small volume ionization chambers, PTW 31022 3D PinPoint and PTW 31023 PinPoint. These two chambers have almost equal cavity volumes, however, they differ considerably in the ratios $d_{L}/d_{R}$; PTW 31022 3D PinPoint chamber has equal cavity dimensions in its longitudinal and radial axis, $d_{L}=d_{R}=2.9\;\text{mm}$, whereas PTW 31023 PinPoint chamber is more elongated, having $d_{L}=5.0\;\text{mm}$ and $d_{R}=2.0\;\text{mm}$ (see also Table 1).

### 2.5 Uncertainty assessment and statistical analysis

Measurement uncertainties were estimated following the recommendations of the *Evaluation of measurement data* —* Guide to the expression of uncertainty in measurement* and from IAEA publications TECDOC‐1585 and TRS‐398.^39^, ^40^, ^41^

#### 2.5.1 Field size uncertainty

For a given field size, the uncertainty of a clinical field size was calculated as the uncertainty of the clinical field dimensions measured with EBT3 films combined with the uncertainty of the reproducibility of the nominal field size setup.

The Type A uncertainty of field dimensions was found negligible, while the Type B uncertainty was estimated as 0.07 mm assuming an uniform probability density function given by the pixel resolution of film measurements.

The variation of field size setup was obtained by setting and resetting a particular field, that is, opening collimators to their maximal values and then setting the collimators to a certain field and measuring lateral beam profiles in a 3D water phantom to obtain differences in measured field sizes. This procedure was repeated several times for the two smallest fields 0.5 and 0.8 cm before each measurement session. We found that the measured field sizes were always within the interval ±0.15 mm. We assumed that differences between measured field sizes (for a single field) have approximately normal distribution with 95% of values inside limits L=±0.1mm. Hence, the uncertainty of the reproducibility of the nominal field size setup was estimated as $u_{B,x}=L/2=0.05\;\text{mm}$.

The combination of both uncertainties yielded a total uncertainty of 0.09 mm for the clinical field sizes $S_{\mathrm{clin}}$.

#### 2.5.2 Field output factors

For the determination of field output factors, signals measured with EBT3 films and W1 plastic scintillator were used jointly after normalization and rescaling. Detailed methodology is described in the first part of our study.^32^

For EBT3 films, the total uncertainty budget includes several sources of uncertainty: intra‐fragment uncertainty, inter‐fragment uncertainty, and intra‐lot difference with respect to the calibration. Estimated total relative uncertainty was 1.7% for measurements made on the Elekta linac and 2.2% for measurements made on the Varian linac.

Dispersion of measured signals and uncertainties associated with the calibration procedure were considered as type A uncertainty for measurements with W1 plastic scintillator. Total uncertainties ranged from 0.3% to 1.1% and from 0.8% to 1.3% for measurements made on the Elekta and Varian linacs, respectively.

Mean values of the signals measured with both detectors and associated uncertainties were employed in the fitting to the analytical function from Eq. (7). Field output factor uncertainties were obtained with parametric bootstrap resampling and were highest for the smallest investigated field of 0.5 cm ranging from 1.0% to 1.4% on the Elekta linac and from 1.6% to 2.5% on the Varian linac.^32^

#### 2.5.3 Output correction factors

For the determination of output correction factors, field output factors $\Omega \left(S_{\mathrm{clin}}\right)$ obtained from the analytical function from Eq. (7) were used together with ratios $M_{Q_{\mathrm{clin}}}^{f_{\mathrm{clin}}}/M_{Q_{\mathrm{ref}}}^{f_{\mathrm{ref}}}$ between mean values of chamber readings in a particular clinical field $S_{\mathrm{clin}}$ and mean values of chamber readings in the reference field, as shown in Eq. (6). In addition to type A uncertainty, type B uncertainty of the signal readings was estimated from the uncertainty of the field size setup. Estimated uncertainty of the field size reproducibility, denoted as $u_{B,x}$ was transposed to the uncertainty of the measured signal, denoted as $u_{B,y}$. Transposition of the uncertainty of $S_{\mathrm{clin}}$ (x‐axis) to the uncertainty of the measured signal $M_{Q_{\mathrm{clin}}}^{f_{\mathrm{clin}}}$ (y‐axis) has been done as

(10) $$\begin{array}{c} u_{B}=u_{B,y}=u_{B,x}\cdot \frac{d}{dS_{\mathrm{clin}}}\Omega \left(S_{\mathrm{clin}}\right) \end{array}$$

Combined uncertainty $u_{C}$ of the mean value of measured signals for particular clinical field $S_{\mathrm{clin}}$ was finally estimated as the combination in quadrature of both types of uncertainties. No other influencing quantities were considered as contributing factors to the type B uncertainty including possible field size‐dependent effects such as polarity, recombination, or stem effect. In our opinion, inclusion of additional uncertainties of type B could lead to an overestimation of the overall uncertainty budget, since our approach was to determine total output correction factors, which contain all individual correction factors.

A combination of the uncertainty of type A and estimated uncertainty of type B, $u_{B,x}=L/2=0.05\text{mm}$(L=±0.1mm), yielded the overall relative uncertainties $u_{C}\left(k=1\right)$ around 1.0%, ranging from 0.7 to 1.3% for different $M_{Q_{\mathrm{clin}}}^{f_{\mathrm{clin}}}/M_{Q_{\mathrm{ref}}}^{f_{\mathrm{ref}}}$ ratios.

#### 2.5.4 Comparison with data given in TRS‐483

Values of output correction factors provided in TRS‐483 were compared with the data obtained in our study for three ionization chambers: IBA CC04, PTW 31016 PinPoint 3D, and SI Exradin A16. Comparison was done only for perpendicular orientation of ionization chambers since the TRS‐483 does not give data for parallel orientation and for field sizes for which detector‐specific output corrections do not exceed 5%.

For TRS‐483, values for output correction factors and their estimated uncertainties were taken from tables 26, 27, and 37 (pages 131, 134, and 193 in the TRS‐483). Before comparison with our results, those values were interpolated to match clinical field sizes $S_{\mathrm{clin}}$ from our study. Compatibility of both sets was evaluated using the difference between corresponding values of output correction factors from both datasets, that is, $k_{TRS-483}-k_{\mathrm{perp}}$, where $k_{TRS-483}$ denotes output correction factors from TRS‐483, and $k_{\mathrm{perp}}$ stands for the output correction factors obtained in our study in perpendicular orientation of ionization chambers. We assumed that the output correction factors between values in the TRS‐483 and our data would differ significantly (within 95% confidence limits), if the relation

(11) $$\begin{array}{c} \left|k_{TRS-483}-k_{\mathrm{perp}}\right|>u_{C}\left(k=2\right) \end{array}$$

is satisfied.

#### 2.5.5 Comparison between two orientations

Differences between values of output correction factors determined for perpendicular and parallel orientation of ionization chambers in the beam were evaluated by using the ratio $k_{\mathrm{perp}}/k_{\mathrm{para}}$ from Eq. (9) together with corresponding combined uncertainties $u_{C}$ applying coverage factor k = 2. We assumed that output correction factors in perpendicular and parallel orientation differ significantly within 95% confidence limits, on condition that the relation

(12) $$\begin{array}{c} \frac{k_{\mathrm{perp}}}{k_{\mathrm{para}}}-1>u_{C}\left(k=2\right) \end{array}$$

is satisfied.

## 3 Results

### 3.1 Output correction factors

Tables 2 and 3 show total output correction factors $k_{Q_{\mathrm{clin}},Q_{\mathrm{ref}}}^{f_{\mathrm{clin}},f_{\mathrm{ref}}}\left(S_{\mathrm{clin}}\right)$ for seven ionization chambers determined for nine radiation fields in four photon beams obtained from Elekta Versa HD linac. Data are presented for two alternative orientations of ionization chambers in the beam, perpendicular, and parallel. For every detector, the values of $k_{Q_{\mathrm{clin}},Q_{\mathrm{ref}}}^{f_{\mathrm{clin}},f_{\mathrm{ref}}}$ for perpendicular orientation can be directly compared to the corresponding values given in TRS‐483.

**Table 2:** Output correction factors $k_{Q_{\mathrm{clin}},Q_{\mathrm{ref}}}^{f_{\mathrm{clin}},f_{\mathrm{ref}}}$ obtained on Elekta Versa HD linac for seven ionization chambers and four investigated photon beams for perpendicular orientation as described in the text (see Fig. 1(a)). These values were obtained by using Eq. (6) for nine equivalent square small field sizes $S_{\mathrm{clin}}$. Values in brackets show absolute uncertainties (1 SD) in the last one or two digits. Measured data represent “total” correction factors, including volume averaging effect as well as perturbation correction factors. Values for output correction factors which are greater than 5% of unity are displayed in bold‐face type.

Energy	$S_{\mathrm{clin}}$ (cm)	IBA CC04	IBA Razor IC	PTW 31016 3D PinPoint	PTW 31021 3D Semiflex	PTW 31022 3D PinPoint	PTW 31023 PinPoint	SI Exradin A16
6 MV WFF	0.60	**1.245 (19)**	**1.119 (17)**	**1.199 (18)**	**1.461 (25)**	**1.131 (18)**	**1.198 (20)**	**1.152 (17)**
	0.87	**1.104 (9)**	1.036 (9)	**1.099 (9)**	**1.157 (10)**	1.047 (10)	**1.074 (9)**	**1.073 (9)**
	1.03	**1.054 (7)**	1.017 (7)	**1.059 (7)**	**1.077 (8)**	1.020 (7)	1.026 (7)	1.042 (7)
	1.51	1.011 (6)	1.001 (6)	1.020 (6)	1.020 (6)	1.005 (6)	1.007 (6)	1.015 (7)
	2.04	1.004 (6)	0.998 (7)	1.008 (6)	1.008 (6)	0.997 (6)	1.004 (6)	1.010 (7)
	3.06	1.002 (6)	1.001 (6)	1.003 (6)	1.001 (6)	0.996 (6)	0.999 (6)	1.007 (6)
	4.04	1.000 (6)	1.002 (6)	1.002 (6)	0.999 (6)	0.995 (6)	0.998 (6)	1.004 (6)
	5.04	1.000 (6)	1.004 (6)	1.004 (6)	1.000 (6)	0.996 (6)	0.998 (6)	1.003 (6)
	10.04	1.001 (0)	1.001 (1)	1.001 (0)	1.001 (0)	1.001 (0)	1.001 (1)	1.001 (2)
6 MV FFF	0.59	**1.267 (22)**	**1.096 (18)**	**1.180 (20)**	**1.427 (26)**	**1.142 (22)**	**1.187 (21)**	**1.143 (19)**
	0.85	**1.092 (11)**	1.025 (10)	**1.070 (11)**	**1.124 (12)**	**1.055 (11)**	**1.075 (11)**	1.048 (11)
	1.03	1.049 (9)	1.015 (9)	1.042 (9)	**1.072 (9)**	1.034 (9)	1.034 (9)	1.035 (9)
	1.52	1.010 (8)	1.003 (8)	1.017 (8)	1.018 (8)	1.007 (8)	1.010 (8)	1.016 (8)
	2.03	0.998 (8)	0.992 (8)	1.003 (8)	1.001 (8)	0.996 (8)	1.000 (8)	1.009 (8)
	3.04	0.994 (7)	0.995 (7)	0.996 (7)	0.994 (7)	0.991 (7)	0.991 (7)	1.006 (7)
	4.03	0.996 (7)	0.998 (7)	0.999 (7)	0.995 (7)	0.992 (7)	0.994 (7)	1.006 (7)
	5.01	0.997 (8)	0.999 (8)	0.999 (8)	0.996 (8)	0.995 (8)	0.995 (8)	1.006 (8)
	9.94	0.999 (0)	0.999 (0)	0.999 (0)	0.999 (0)	0.999 (0)	0.999 (0)	0.999 (0)
10 MV WFF	0.62	**1.230 (19)**	**1.099 (17)**	**1.211 (19)**	**1.499 (26)**	**1.167 (21)**	**1.265 (21)**	**1.143 (18)**
	0.87	**1.085 (10)**	1.017 (9)	**1.070 (10)**	**1.135 (11)**	**1.057 (11)**	**1.061 (10)**	1.042 (11)
	1.06	1.053 (9)	1.009 (8)	**1.051 (9)**	**1.079 (9)**	1.011 (9)	1.034 (8)	1.020 (8)
	1.55	1.012 (7)	0.996 (7)	1.020 (7)	1.024 (7)	1.005 (8)	1.011 (7)	1.008 (7)
	2.05	0.998 (7)	0.989 (7)	1.000 (7)	1.004 (7)	0.993 (7)	0.997 (7)	1.002 (7)
	3.08	0.994 (6)	0.992 (6)	0.993 (6)	0.994 (6)	0.988 (6)	0.989 (6)	0.999 (6)
	4.06	0.994 (6)	0.995 (6)	0.995 (6)	0.993 (6)	0.990 (6)	0.992 (6)	0.999 (6)
	5.05	0.996 (7)	0.998 (7)	0.997 (7)	0.995 (7)	0.993 (7)	0.995 (7)	1.000 (7)
	10.05	1.001 (1)	1.001 (0)	1.001 (0)	1.001 (1)	1.001 (1)	1.001 (0)	1.001 (1)
10 MV FFF	0.58	**1.202 (20)**	**1.061 (17)**	**1.126 (18)**	**1.425 (24)**	**1.118 (18)**	**1.148 (19)**	**1.098 (17)**
	0.86	**1.065 (11)**	1.011 (11)	**1.063 (11)**	**1.109 (12)**	**1.051 (11)**	**1.053 (11)**	1.038 (11)
	1.04	1.042 (10)	1.009 (9)	1.042 (10)	**1.068 (10)**	1.020 (10)	1.027 (11)	1.027 (9)
	1.52	1.009 (8)	0.997 (8)	1.016 (8)	1.020 (8)	1.002 (8)	1.009 (8)	1.011 (8)
	2.04	1.004 (8)	0.993 (8)	1.003 (8)	1.005 (8)	0.996 (8)	1.002 (8)	1.009 (8)
	3.02	0.995 (7)	0.993 (7)	0.994 (7)	0.996 (7)	0.989 (7)	0.990 (7)	1.002 (7)
	4.01	0.998 (7)	0.998 (7)	0.997 (7)	0.998 (7)	0.992 (7)	0.994 (7)	1.003 (7)
	4.99	1.001 (8)	1.001 (8)	0.999 (8)	1.000 (8)	0.997 (8)	0.997 (8)	1.005 (8)
	9.90	0.999 (1)	0.999 (0)	0.999 (0)	0.999 (1)	0.999 (1)	0.999 (0)	0.999 (0)

**Table 3:** Output correction factors $k_{Q_{\mathrm{clin}},Q_{\mathrm{ref}}}^{f_{\mathrm{clin}},f_{\mathrm{ref}}}$ obtained on Elekta Versa HD linac for seven ionization chambers and four investigated photon beams for parallel orientation as described in the text (see Fig. 1(b)). These values were obtained by using Eq. (6) for nine equivalent square small field sizes $S_{\mathrm{clin}}$. Values in brackets show absolute uncertainties (1 SD) in the last one or two digits. Measured data represent “total” correction factors, including volume averaging effect as well as perturbation correction factors. Values for output correction factors which are greater than 5% of unity are displayed in bold‐face type.

Energy	$S_{\mathrm{clin}}$ (cm)	IBA CC04	IBA Razor IC	PTW 31016 3D PinPoint	PTW 31021 3D Semiflex	PTW 31022 3D PinPoint	PTW 31023 PinPoint	SI Exradin A16
6 MV WFF	0.60	**1.223 (19)**	1.037 (15)	**1.096 (16)**	**1.394 (23)**	1.043 (15)	1.016 (14)	**1.112 (16)**
	0.87	**1.090 (9)**	1.012 (9)	1.047 (9)	**1.145 (10)**	1.029 (9)	1.012 (8)	1.049 (9)
	1.03	1.046 (7)	1.003 (7)	1.024 (7)	**1.075 (8)**	1.009 (7)	1.000 (8)	1.022 (7)
	1.51	1.009 (6)	1.001 (6)	1.007 (6)	1.021 (6)	1.002 (6)	1.001 (6)	1.009 (6)
	2.04	1.003 (6)	1.005 (7)	1.004 (6)	1.007 (6)	1.002 (6)	1.003 (6)	1.008 (7)
	3.06	1.002 (6)	1.005 (6)	1.003 (6)	1.000 (6)	1.000 (6)	1.001 (6)	1.007 (6)
	4.04	0.999 (6)	1.004 (6)	1.000 (6)	0.998 (6)	0.998 (6)	1.000 (6)	1.005 (6)
	5.04	1.000 (6)	1.005 (6)	1.000 (6)	0.999 (6)	0.998 (6)	0.999 (6)	1.004 (6)
	10.04	1.001 (0)	1.001 (2)	1.001 (0)	1.001 (1)	1.001 (0)	1.001 (0)	1.001 (2)
6 MV FFF	0.59	**1.222 (21)**	1.019 (16)	**1.085 (18)**	**1.397 (25)**	**1.100 (18)**	1.047 (17)	**1.075 (18)**
	0.85	**1.073 (11)**	1.005 (10)	**1.054 (11)**	**1.117 (11)**	1.033 (10)	1.008 (10)	1.026 (10)
	1.03	1.044 (9)	1.001 (9)	1.022 (9)	**1.071 (9)**	1.016 (9)	1.002 (9)	1.019 (9)
	1.52	1.009 (8)	0.998 (8)	1.005 (8)	1.017 (8)	1.002 (8)	1.000 (8)	1.012 (8)
	2.03	0.999 (8)	0.996 (8)	0.998 (8)	1.003 (8)	0.997 (8)	0.996 (8)	1.007 (8)
	3.04	0.994 (7)	0.995 (7)	0.996 (7)	0.995 (7)	0.994 (7)	0.994 (7)	1.006 (7)
	4.03	0.995 (7)	0.997 (7)	0.997 (7)	0.994 (7)	0.995 (7)	0.996 (7)	1.006 (7)
	5.01	0.996 (8)	0.998 (8)	0.998 (8)	0.996 (8)	0.996 (8)	0.995 (8)	1.007 (8)
	9.94	0.999 (0)	0.999 (0)	0.999 (0)	0.999 (1)	0.999 (0)	0.999 (0)	0.999 (1)
10 MV WFF	0.62	**1.207 (19)**	1.010 (15)	**1.104 (17)**	**1.386 (23)**	**1.063 (16)**	1.012 (15)	**1.082 (17)**
	0.87	**1.064 (10)**	0.989 (9)	1.037 (9)	**1.123 (10)**	1.023 (10)	1.002 (11)	1.017 (9)
	1.06	1.039 (8)	0.991 (8)	1.015 (8)	**1.076 (9)**	1.008 (8)	0.999 (8)	1.012 (8)
	1.55	1.011 (7)	0.994 (7)	1.004 (7)	1.024 (7)	1.001 (7)	0.998 (7)	1.003 (7)
	2.05	1.000 (7)	0.995 (7)	0.997 (7)	1.005 (7)	0.995 (7)	0.993 (7)	0.996 (7)
	3.08	0.994 (6)	0.995 (6)	0.994 (6)	0.994 (6)	0.993 (6)	0.991 (6)	0.998 (6)
	4.06	0.994 (6)	0.996 (6)	0.994 (6)	0.993 (6)	0.993 (6)	0.993 (6)	0.998 (6)
	5.05	0.996 (7)	0.996 (7)	0.996 (7)	0.995 (7)	0.995 (7)	0.995 (7)	1.000 (7)
	10.05	1.001 (1)	1.001 (1)	1.001 (1)	1.001 (0)	1.001 (0)	1.001 (0)	1.001 (1)
10 MV FFF	0.58	**1.171 (19)**	0.981 (15)	**1.072 (17)**	**1.331 (22)**	1.043 (17)	1.016 (16)	**1.056 (17)**
	0.86	1.047 (11)	0.983 (10)	1.038 (11)	**1.097 (12)**	1.022 (11)	1.005 (11)	1.011 (11)
	1.04	1.022 (9)	0.992 (9)	1.023 (10)	**1.064 (10)**	0.997 (9)	0.995 (10)	1.011 (9)
	1.52	1.006 (8)	0.989 (8)	1.005 (8)	1.019 (8)	0.996 (8)	0.997 (8)	1.002 (8)
	2.04	1.002 (8)	0.993 (8)	1.003 (8)	1.007 (8)	0.997 (8)	0.996 (8)	1.002 (8)
	3.02	0.995 (7)	0.992 (7)	0.997 (7)	0.995 (7)	0.991 (7)	0.993 (7)	1.000 (7)
	4.01	0.998 (7)	0.995 (7)	1.001 (8)	0.997 (7)	0.994 (7)	0.995 (7)	1.004 (7)
	4.99	1.001 (8)	1.000 (8)	1.002 (8)	0.999 (8)	0.998 (8)	0.999 (8)	1.007 (8)
	9.90	0.999 (1)	0.999 (0)	0.999 (2)	0.999 (0)	0.999 (0)	0.999 (0)	0.999 (0)

**Figure 2:**
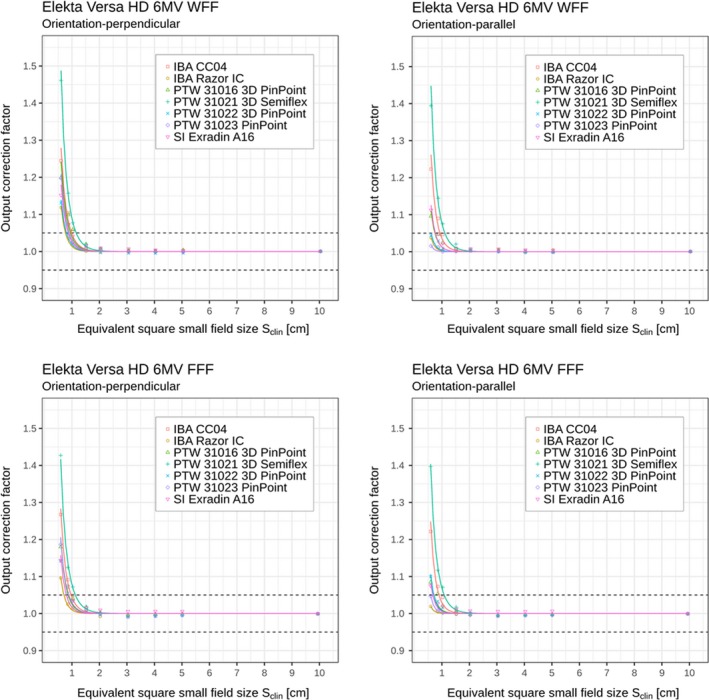
Detector‐specific output correction factors for seven ionization chambers for 6 MV WFF and FFF beams; data are for perpendicular and parallel orientations measured on Elekta Versa HD linac. Output correction factors are presented as individual values/points $k_{Q_{\mathrm{clin}},Q_{\mathrm{ref}}}^{f_{\mathrm{clin}},f_{\mathrm{ref}}}$ (shown in the plots as discrete data points) and as analytical functions $k\left(S_{\mathrm{clin}}\right)$ (shown in the plots by the solid curves) applying Eqs. (6) and (8), respectively. Displayed data represent “total” correction factors and include contributions from both volume averaging effect as well as perturbation correction factors. Horizontal dashed lines represent limits (0.95–1.05) within which correction factors are recommended. [Color figure can be viewed at http://www.wileyonlinelibrary.com]

**Figure 3:**
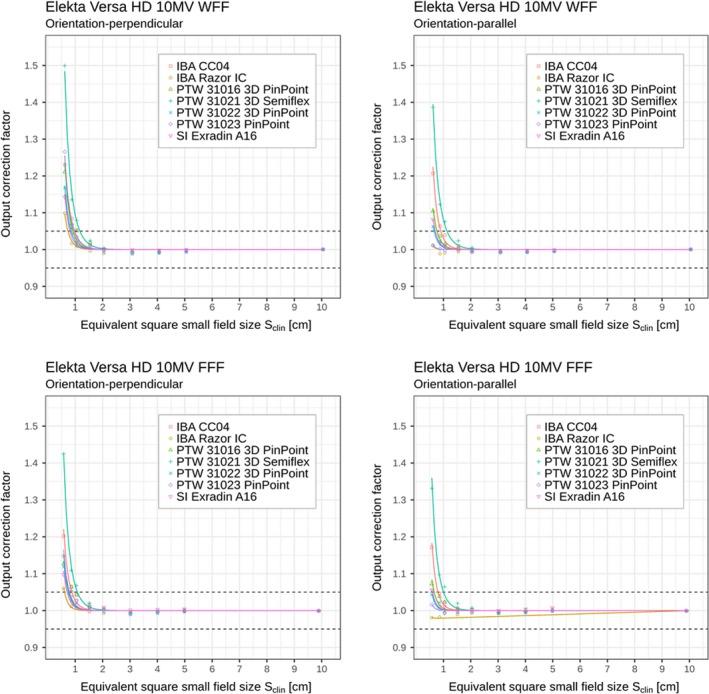
Detector‐specific output correction factors for seven ionization chambers for 10 MV WFF and FFF beams; data are for perpendicular and parallel orientations measured on Elekta Versa HD linac. Output correction factors are presented as individual values/points $k_{Q_{\mathrm{clin}},Q_{\mathrm{ref}}}^{f_{\mathrm{clin}},f_{\mathrm{ref}}}$ (shown in the plots as discrete data points) and as analytical functions $k\left(S_{\mathrm{clin}}\right)$ (shown in the plots by the solid curves) applying Eqs. (6) and (8), respectively. Displayed data represent “total” correction factors and include contributions from both volume averaging effect as well as perturbation correction factors. Horizontal dashed lines represent limits (0.95–1.05) within which correction factors are recommended. [Color figure can be viewed at http://www.wileyonlinelibrary.com]

For graphical presentation, experimentally determined values of output correction factors were fitted with the analytical function given by Eq. (8). Graphs of analytical functions for $k\left(S_{\mathrm{clin}}\right)$ vs $S_{\mathrm{clin}}$ for all ionization chambers and beam energies and for both perpendicular and parallel orientations on Elekta Versa HD linac are displayed in Figs. 2 and 3. Solid curves in these figures represent fits to the data points using the analytic function $k\left(S_{\mathrm{clin}}\right)$ given by Eq. (8). The data points obtained from Eq. (6) were fitted down to the smallest field size of 0.5 cm^2^ × 0.5 cm^2^.

**Figure 4:**
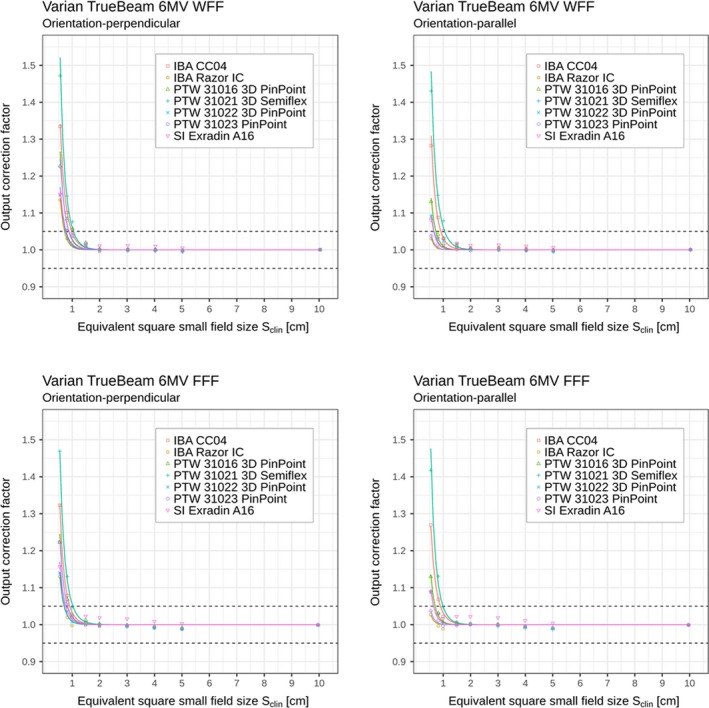
Detector‐specific output correction factors for seven ionization chambers for 6 MV WFF and FFF beams; data are for perpendicular and parallel orientations measured on Varian TrueBeam linac. Output correction factors are presented as individual values/points $k_{Q_{\mathrm{clin}},Q_{\mathrm{ref}}}^{f_{\mathrm{clin}},f_{\mathrm{ref}}}$ (shown in the plots as discrete data points) and as analytical functions $k\left(S_{\mathrm{clin}}\right)$ (shown in the plots by the solid curves) applying Eqs. (6) and (8), respectively. Displayed data represent “total” correction factors and include contributions from both, volume averaging effect as well as perturbation correction factors. Horizontal dashed lines represent limits (0.95–1.05) within which correction factors are recommended. [Color figure can be viewed at http://www.wileyonlinelibrary.com]

**Figure 5:**
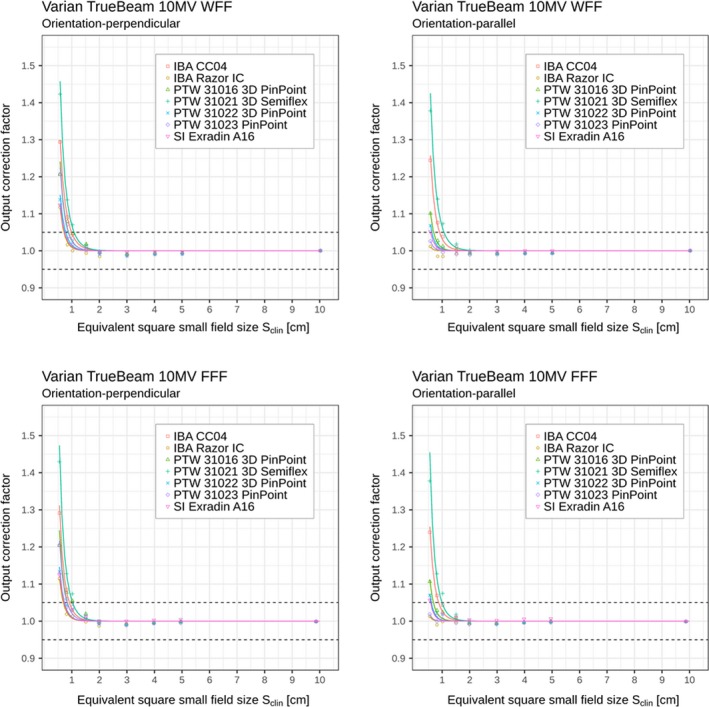
Detector‐specific output correction factors for seven ionization chambers for 10 MV WFF and FFF beams; data are for perpendicular and parallel orientations measured on Varian TrueBeam linac. Output correction factors are presented as individual values/points $k_{Q_{\mathrm{clin}},Q_{\mathrm{ref}}}^{f_{\mathrm{clin}},f_{\mathrm{ref}}}$ (shown in the plots as discrete data points) and as analytical functions $k\left(S_{\mathrm{clin}}\right)$ (shown in the plots by the solid curves) applying Eqs. (6) and (8), respectively. Displayed data represent “total” correction factors and include contributions from both, volume averaging effect as well as perturbation correction factors. Horizontal dashed lines represent limits (0.95–1.05) within which correction factors are recommended. [Color figure can be viewed at http://www.wileyonlinelibrary.com]

Similarly as for Elekta Versa HD linac, Tables 4 and 5 and Figs. 4 and 5 show the corresponding output correction factors determined on Varian TrueBeam linac for different combinations of beam energies, ionization chambers, and orientations of the chamber axis with respect to the beam axis.

**Table 4:** Output correction factors $k_{Q_{\mathrm{clin}},Q_{\mathrm{ref}}}^{f_{\mathrm{clin}},f_{\mathrm{ref}}}$ obtained on Varian TrueBeam linac for seven ionization chambers and four investigated photon beams for perpendicular orientation as described in the text (see Fig. 1(a)). These values were obtained by using Eq. (6) for nine equivalent square small field sizes $S_{\mathrm{clin}}$. Values in brackets show absolute uncertainties (1 SD) in the last one or two digits. Measured data represent “total” correction factors, including volume averaging effect as well as perturbation correction factors. Values for output correction factors which are greater than 5% of unity are displayed in bold‐face type.

Energy	$S_{\mathrm{clin}}$ (cm)	IBA CC04	IBA Razor IC	PTW 31016 3D PinPoint	PTW 31021 3D Semiflex	PTW 31022 3D PinPoint	PTW 31023 PinPoint	SI Exradin A16
6 MV WFF	0.56	**1.335 (31)**	**1.135 (25)**	**1.228 (28)**	**1.472 (35)**	**1.148 (25)**	**1.226 (28)**	**1.151 (26)**
	0.81	**1.101 (15)**	1.031 (14)	**1.084 (15)**	**1.146 (16)**	**1.054 (14)**	**1.080 (15)**	**1.051 (14)**
	1.01	**1.054 (13)**	1.020 (12)	**1.057 (13)**	**1.076 (13)**	1.035 (12)	1.047 (13)	1.040 (12)
	1.50	1.012 (11)	1.003 (11)	1.020 (11)	1.016 (11)	1.010 (11)	1.010 (11)	1.018 (11)
	2.00	1.000 (11)	0.995 (11)	1.003 (12)	1.002 (12)	0.999 (11)	1.000 (11)	1.011 (12)
	3.03	1.001 (11)	1.001 (11)	1.001 (11)	0.999 (11)	0.999 (11)	0.998 (11)	1.012 (11)
	4.03	1.000 (11)	1.002 (11)	1.001 (11)	0.998 (11)	1.000 (11)	0.997 (11)	1.010 (11)
	5.02	0.997 (10)	0.999 (10)	0.998 (10)	0.996 (10)	0.998 (10)	0.995 (10)	1.005 (10)
	10.03	1.001 (1)	1.001 (0)	1.001 (1)	1.001 (0)	1.001 (1)	1.001 (0)	1.001 (0)
6 MV FFF	0.54	**1.323 (33)**	**1.130 (27)**	**1.222 (30)**	**1.469 (38)**	**1.140 (28)**	**1.224 (30)**	**1.157 (28)**
	0.82	**1.079 (17)**	1.019 (16)	**1.069 (17)**	**1.130 (18)**	1.035 (16)	**1.060 (17)**	1.050 (16)
	0.99	1.024 (15)	0.997 (14)	1.029 (15)	1.047 (15)	1.007 (15)	1.019 (15)	1.025 (15)
	1.49	1.004 (14)	0.999 (14)	1.012 (14)	1.008 (14)	1.000 (14)	1.003 (14)	1.022 (14)
	1.99	0.999 (14)	0.996 (14)	1.003 (14)	1.000 (14)	0.996 (14)	1.000 (14)	1.019 (14)
	3.00	0.997 (13)	0.998 (13)	0.999 (13)	0.998 (13)	0.995 (13)	0.995 (13)	1.016 (13)
	3.99	0.993 (12)	0.995 (12)	0.995 (12)	0.991 (12)	0.991 (12)	0.991 (12)	1.009 (12)
	5.00	0.989 (11)	0.990 (11)	0.991 (11)	0.988 (11)	0.988 (11)	0.988 (11)	1.003 (11)
	9.96	0.999 (0)	0.999 (1)	0.999 (0)	0.999 (0)	0.999 (0)	0.999 (0)	0.999 (1)
10 MV WFF	0.57	**1.294 (36)**	**1.099 (17)**	**1.206 (33)**	**1.423 (40)**	**1.138 (31)**	**1.207 (33)**	**1.124 (30)**
	0.84	**1.091 (17)**	1.017 (9)	**1.079 (16)**	**1.137 (17)**	**1.051 (16)**	**1.072 (16)**	1.033 (16)
	1.03	1.046 (15)	1.009 (8)	1.047 (15)	**1.070 (15)**	1.025 (14)	1.034 (14)	1.017 (14)
	1.52	1.010 (13)	0.996 (7)	1.018 (13)	1.016 (13)	1.001 (13)	1.004 (13)	1.006 (13)
	2.01	0.995 (13)	0.989 (7)	0.997 (13)	0.998 (13)	0.991 (13)	0.993 (13)	0.998 (13)
	3.00	0.991 (12)	0.992 (6)	0.991 (12)	0.991 (12)	0.986 (12)	0.987 (12)	0.996 (12)
	4.02	0.993 (12)	0.995 (6)	0.994 (12)	0.992 (12)	0.990 (12)	0.991 (12)	0.998 (12)
	5.01	0.994 (11)	0.998 (7)	0.994 (11)	0.993 (11)	0.991 (11)	0.992 (11)	0.998 (11)
	10.02	1.000 (0)	1.001 (0)	1.000 (0)	1.000 (0)	1.000 (0)	1.000 (0)	1.000 (0)
10 MV FFF	0.55	**1.291 (25)**	**1.118 (30)**	**1.204 (23)**	**1.430 (28)**	**1.133 (21)**	**1.205 (23)**	**1.125 (21)**
	0.81	**1.085 (14)**	1.016 (15)	**1.077 (14)**	**1.128 (14)**	1.044 (13)	**1.060 (13)**	1.037 (13)
	1.02	1.050 (12)	1.000 (14)	**1.055 (12)**	**1.074 (12)**	1.031 (12)	1.039 (12)	1.030 (12)
	1.51	1.012 (10)	0.993 (13)	1.020 (10)	1.017 (10)	1.006 (10)	1.008 (10)	1.012 (10)
	1.99	0.996 (10)	0.985 (12)	0.998 (10)	0.998 (10)	0.993 (10)	0.994 (10)	1.001 (10)
	2.98	0.992 (9)	0.989 (12)	0.992 (9)	0.992 (9)	0.990 (9)	0.988 (9)	1.000 (9)
	3.98	0.996 (9)	0.994 (12)	0.997 (9)	0.995 (9)	0.994 (9)	0.994 (9)	1.003 (9)
	4.96	0.998 (9)	0.995 (11)	0.999 (9)	0.998 (9)	0.997 (9)	0.995 (9)	1.005 (9)
	9.87	0.999 (0)	1.000 (1)	0.999 (0)	0.999 (0)	0.999 (0)	0.999 (0)	0.999 (1)

**Table 5:** Output correction factors $k_{Q_{\mathrm{clin}},Q_{\mathrm{ref}}}^{f_{\mathrm{clin}},f_{\mathrm{ref}}}$ obtained on Varian TrueBeam linac for seven ionization chambers and four investigated photon beams for parallel orientation as described in the text (see Fig. 1(b)). These values were obtained by using Eq. (6) for nine equivalent square small field sizes $S_{\mathrm{clin}}$. Values in brackets show absolute uncertainties (1 SD) in the last one or two digits. Measured data represent “total” correction factors including volume averaging effect as well as perturbation correction factors. Values for output correction factors which are greater than 5% of unity are displayed in bold‐face type.

Energy	$S_{\mathrm{clin}}$ (cm)	IBA CC04	IBA Razor IC	PTW 31016 3D PinPoint	PTW 31021 3D Semiflex	PTW 31022 3D PinPoint	PTW 31023 PinPoint	SI Exradin A16
6 MV WFF	0.56	**1.283 (29)**	1.030 (22)	**1.131 (25)**	**1.430 (34)**	**1.090 (24)**	1.038 (23)	**1.081 (24)**
	0.81	**1.088 (15)**	1.006 (14)	1.042 (14)	**1.148 (16)**	1.032 (14)	1.016 (14)	1.031 (14)
	1.01	**1.052 (12)**	1.012 (12)	1.031 (12)	**1.079 (13)**	1.024 (12)	1.016 (12)	1.031 (12)
	1.50	1.014 (11)	1.007 (11)	1.011 (11)	1.017 (11)	1.003 (11)	1.001 (11)	1.018 (11)
	2.00	1.004 (12)	1.004 (12)	1.003 (12)	1.003 (12)	0.999 (11)	0.998 (11)	1.012 (12)
	3.03	1.003 (11)	1.007 (11)	1.003 (11)	1.000 (11)	1.000 (11)	1.000 (11)	1.014 (11)
	4.03	1.001 (11)	1.005 (11)	1.001 (11)	0.999 (11)	0.998 (11)	0.998 (11)	1.010 (11)
	5.02	0.998 (10)	1.001 (10)	0.998 (10)	0.996 (10)	0.996 (10)	0.996 (10)	1.007 (10)
	10.03	1.001 (0)	1.001 (0)	1.001 (0)	1.001 (0)	1.001 (0)	1.001 (1)	1.001 (0)
6 MV FFF	0.54	**1.270 (31)**	1.027 (25)	**1.130 (27)**	**1.418 (35)**	**1.088 (26)**	1.037 (25)	**1.089 (26)**
	0.82	**1.068 (17)**	0.996 (16)	1.031 (16)	**1.131 (18)**	1.029 (16)	1.012 (16)	1.029 (16)
	0.99	1.022 (15)	0.989 (14)	1.008 (15)	1.048 (15)	1.003 (15)	0.996 (14)	1.017 (15)
	1.49	1.006 (14)	1.000 (14)	1.004 (14)	1.006 (14)	0.999 (14)	0.998 (14)	1.022 (14)
	1.99	1.002 (14)	1.003 (14)	1.002 (14)	1.000 (14)	1.000 (14)	1.001 (14)	1.022 (14)
	3.00	0.998 (13)	1.002 (13)	1.000 (13)	0.995 (13)	0.999 (13)	0.999 (13)	1.019 (13)
	3.99	0.993 (12)	0.996 (12)	0.995 (12)	0.991 (12)	0.994 (12)	0.994 (12)	1.011 (12)
	5.00	0.990 (11)	0.992 (11)	0.991 (11)	0.986 (11)	0.990 (11)	0.991 (11)	1.004 (11)
	9.96	0.999 (0)	0.999 (1)	0.999 (0)	0.999 (0)	0.999 (0)	0.999 (1)	0.999 (0)
10 MV WFF	0.57	**1.244 (34)**	1.012 (27)	**1.101 (29)**	**1.378 (38)**	**1.068 (28)**	1.026 (27)	**1.052 (28)**
	0.84	**1.076 (16)**	0.985 (15)	1.027 (16)	**1.140 (17)**	1.022 (15)	1.004 (15)	1.008 (15)
	1.03	1.038 (15)	0.985 (14)	1.012 (14)	**1.073 (15)**	1.008 (14)	0.996 (14)	1.003 (14)
	1.52	1.010 (13)	0.994 (13)	1.003 (13)	1.018 (13)	0.996 (13)	0.990 (13)	1.001 (13)
	2.01	0.998 (13)	0.992 (13)	0.995 (13)	1.002 (13)	0.992 (13)	0.989 (13)	0.999 (13)
	3.00	0.993 (12)	0.994 (12)	0.993 (12)	0.993 (12)	0.991 (12)	0.990 (12)	0.999 (12)
	4.02	0.994 (12)	0.996 (12)	0.994 (12)	0.993 (12)	0.993 (12)	0.992 (12)	1.000 (12)
	5.01	0.994 (11)	0.996 (11)	0.994 (11)	0.993 (11)	0.993 (11)	0.993 (11)	1.000 (11)
	10.02	1.000 (0)	1.000 (1)	1.000 (0)	1.000 (0)	1.000 (0)	1.000 (0)	1.000 (0)
10 MV FFF	0.55	1.240 (24)	1.013 (19)	1.107 (21)	1.378 (28)	1.069 (20)	1.019 (19)	1.057 (20)
	0.81	1.069 (14)	0.990 (12)	1.029 (13)	1.127 (14)	1.022 (13)	1.000 (13)	1.015 (13)
	1.02	1.043 (12)	0.999 (11)	1.024 (11)	1.075 (12)	1.019 (11)	1.005 (11)	1.019 (11)
	1.51	1.011 (10)	0.998 (10)	1.007 (10)	1.018 (10)	1.000 (10)	0.995 (9)	1.010 (10)
	1.99	0.998 (10)	0.993 (10)	0.996 (10)	1.001 (10)	0.994 (10)	0.991 (10)	1.004 (10)
	2.98	0.994 (9)	0.995 (9)	0.994 (9)	0.993 (9)	0.992 (9)	0.991 (9)	1.002 (9)
	3.98	0.997 (9)	0.998 (9)	0.997 (9)	0.996 (9)	0.996 (9)	0.996 (9)	1.006 (9)
	4.96	0.998 (9)	1.000 (9)	0.999 (9)	0.997 (9)	0.998 (9)	0.996 (9)	1.007 (9)
	9.87	0.999 (0)	0.999 (0)	0.999 (1)	0.999 (1)	0.999 (1)	0.999 (0)	0.999 (0)

Equivalent square field sizes $S_{\mathrm{clin}}$ presented in the Tables 2, 3, 4, 5 correspond to the nominal field sizes 0.5, 0.8, 1.0, 1.5, 2.0, 3.0, 4.0, 5.0, and 10.0 cm.

### 3.2 Volume averaging considerations

**Figure 6:**
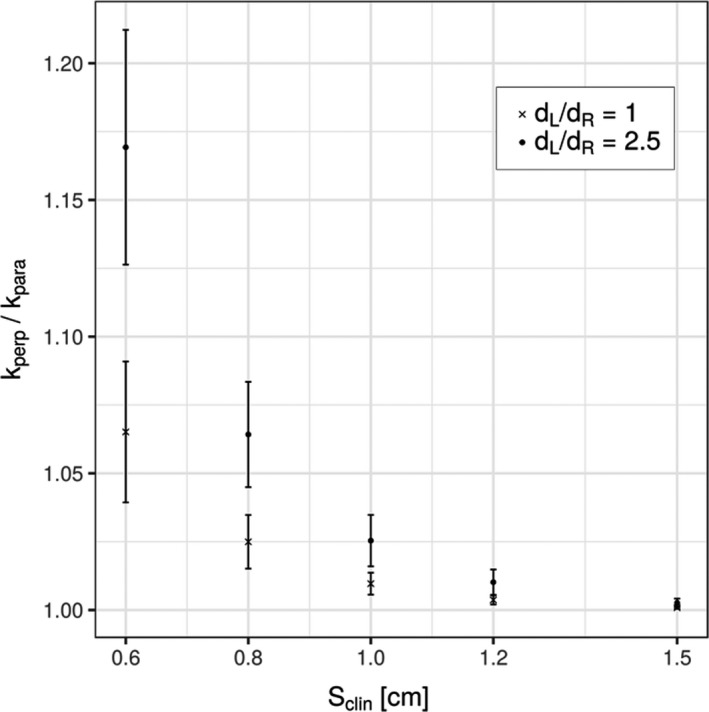
Ratios $k_{\mathrm{perp}}/k_{\mathrm{para}}$ of output correction factors determined in two orientations (perpendicular and parallel) for two ionization chambers, PTW 31022 3D PinPoint and PTW 31023 PinPoint, having different ratios $d_{L}/d_{R}$ between cavity dimension $d_{L}$ in the longitudinal direction and $d_{R}$ in the radial direction, perpendicular to the former. $d_{L}/d_{R}=1$ corresponds to the PTW 31022 3D PinPoint chamber, while $d_{L}/d_{R}=2.5$ corresponds to the PTW 31023 Pinpoint chamber. Average values for output correction factors for all investigated beams were considered for the determination of $k_{\mathrm{perp}}/k_{\mathrm{para}}$ values and were calculated from Eq. (8). Error bars represent the dispersion of data (1 SD).

The plot in Fig. 6 illustrates the dependence of ratios between output correction factors determined in perpendicular and parallel orientations, $k_{\mathrm{perp}}/k_{\mathrm{para}}$, vs equivalent square small field size $S_{\mathrm{clin}}$ for two different ratios $d_{L}/d_{R}$; one set corresponds to the PTW 31022 3D PinPoint chamber ($d_{L}/d_{R}=1$), while the other corresponds to the PTW 31023 Pinpoint chamber ($d_{L}/d_{R}=2.5$). Ratios $k_{\mathrm{perp}}/k_{\mathrm{para}}$ were determined as average values of output correction factors determined in all megavoltage beams on both linear accelerators using the analytical function from Eq. (8).

## 4 Discussion

### 4.1 Comparison with data given in TRS‐483

In TRS‐483 values of output correction factors are provided for nine ionization chambers down to the field sizes for which detector‐specific output correction factors are not greater than ±5%, that is, 0.95 ≤ $k_{Q_{\mathrm{clin}},Q_{\mathrm{ref}}}^{f_{\mathrm{clin}},f_{\mathrm{ref}}}$ ≤1.05.^1^, ^2^ Data for output correction factors for certain combinations of chambers, beam energies, and the smallest field sizes were not given because the uncertainties of these correction factors become large and unreliable.^2^ Since the data for $k_{Q_{\mathrm{clin}},Q_{\mathrm{ref}}}^{f_{\mathrm{clin}},f_{\mathrm{ref}}}$ in the TRS‐483 are given only for perpendicular orientation of the ionization chambers, we did the comparison of output correction factors for this orientation only.

Among the seven ionization chambers from our study set, data for only three chambers are available in TRS‐483 for comparison: IBA CC04, PTW 31016 PinPoint 3D, and SI Exradin A16. To determine the statistical significance of differences between corresponding sets of output correction factors, we have examined fulfillment of the condition from Eq. (11). Data from TRS‐483 were compared with our data for each energy, filtration, and linac. With the exception of only one dataset, no statistically significant differences were found between the two datasets regardless of the beam energy, linac, filtration, or field sizes being evaluated. The only exception among 24 compared datasets was observed for SI Exradin A16 chamber for 0.8 cm^2^ × 0.8 cm^2^ field size in 6 MV WFF beam on Elekta linac, where statistically significant difference based on the condition from Eq. (11) was found between the value of output correction factor published in TRS‐483 and the value obtained in our study. However, also in the latter case, the statistical difference was only marginally significant ($k_{TRS-483}-k_{\mathrm{perp}}=0.036$, while $u_{C}\left(k=2\right)=0.034$). It should be noted that output correction factors were compared for field sizes given in TRS‐483 — for IBA CC04 and PTW 31016 PinPoint 3D chambers only down to 1.0 cm^2^ × 1.0 cm^2^, whereas for SI Exradin A16 chamber, the smallest field in the comparison was 0.8 cm^2^ × 0.8 cm^2^.

We can conclude that with the possible exception stated above, detector‐specific output correction factors obtained in our study for three ionization chambers, IBA CC04, PTW 31016 PinPoint 3D, and SI Exradin A16, confirm the corresponding data published in the TRS‐483 using the 95% confidence limits. The data for output correction factors determined in this experimental study for the remaining four ionization chambers, IBA Razor IC, PTW 31021 3D Semiflex, PTW 31022 PinPoint 3D, and PTW 31023 PinPoint, are considered a valuable supplement to the literature and to the TRS‐483 dataset.

### 4.2 The orientation of ionization chambers

The rationale for recommending perpendicular orientation of ionization chambers in TRS‐483 for the determination of field output factors in small fields has recently been clarified by the authors of TRS‐483.^4^ They have stated that perpendicular orientation was recommended in the TRS‐483 only because at the time of writing the Code of Practice there was a lack of data available for parallel orientation. Indeed, only a few studies reported experimental results for $k_{Q_{\mathrm{clin}},Q_{\mathrm{ref}}}^{f_{\mathrm{clin}},f_{\mathrm{ref}}}$ in small fields both orientations for only a few small or micro ionization chambers.^9^, ^14^, ^19^ Furthermore, lack of homogeneity in data with respect to the size of the normalization field, differences in the definition of field sizes, variations in setup (SSD or SDD used), among others, made the analysis very difficult and unreliable. Therefore, we keep the discussion of the present data within the framework of the results obtained in our study.

In Tables 2, 3, 4, 5 and Figs. 2, 3, 4, 5, it can be seen that for all ionization chambers used in the present study except the IBA CC04 chamber, the output correction factors $k_{Q_{\mathrm{clin}},Q_{\mathrm{ref}}}^{f_{\mathrm{clin}},f_{\mathrm{ref}}}$ are found to be lower for the parallel orientation than the corresponding values determined for the perpendicular orientation. This observation holds for field sizes below 1.0 cm^2^ × 1.0 cm^2^, regardless of the energy or linac being used. The significance of these differences was tested using the condition from Eq. (12). Statistically significant differences within 95% confidence limits were found between output correction factors $k_{Q_{\mathrm{clin}},Q_{\mathrm{ref}}}^{f_{\mathrm{clin}},f_{\mathrm{ref}}}$, determined in both orientations; $k_{Q_{\mathrm{clin}},Q_{\mathrm{ref}}}^{f_{\mathrm{clin}},f_{\mathrm{ref}}}$ values were almost always significantly higher for perpendicular orientation compared to the parallel one for the smallest fields, as shown in Table 6.

**Table 6:** Ratios $k_{\mathrm{perp}}/k_{\mathrm{para}}$ between output correction factors in perpendicular and parallel orientation (see Figs. 1(a) and 1(b)) obtained on Elekta Versa HD and Varian TrueBeam linac for seven ionization chambers and four investigated photon beams. These values were obtained by using Eq. (9). Values in brackets show absolute uncertainties in the last one or two digits using 95% confidence level (k = 2).

Energy	$S_{\mathrm{clin}}$ (cm)	IBA CC04	IBA Razor IC	PTW 31016 3D PinPoint	PTW 31021 3D Semiflex	PTW 31022 3D PinPoint	PTW 31023 PinPoint	SI Exradin A16
Elekta Versa HD								
6 MV WFF	0.60	1.018 (34)	1.078 (32)	1.094 (34)	1.048 (40)	1.084 (36)	1.179 (39)	1.035 (32)
	0.87	1.013 (11)	1.023 (11)	1.050 (11)	1.011 (12)	1.017 (14)	1.061 (12)	1.023 (12)
	1.03	1.008 (6)	1.014 (7)	1.034 (6)	1.002 (7)	1.011 (8)	1.026 (8)	1.019 (7)
	1.51	1.002 (2)	1.000 (4)	1.013 (2)	0.999 (2)	1.002 (3)	1.007 (3)	1.006 (4)
6 MV FFF	0.59	1.038 (33)	1.076 (29)	1.088 (33)	1.022 (37)	1.038 (36)	1.133 (37)	1.064 (31)
	0.85	1.017 (11)	1.020 (10)	1.016 (10)	1.007 (12)	1.021 (11)	1.067 (12)	1.022 (10)
	1.03	1.005 (6)	1.013 (6)	1.019 (7)	1.000 (6)	1.018 (9)	1.033 (8)	1.015 (6)
	1.52	1.001 (2)	1.005 (2)	1.012 (2)	1.001 (2)	1.005 (3)	1.010 (3)	1.004 (2)
10 MV WFF	0.62	1.020 (29)	1.088 (27)	1.096 (30)	1.082 (37)	1.099 (36)	1.251 (35)	1.056 (29)
	0.87	1.019 (12)	1.028 (11)	1.032 (12)	1.011 (12)	1.033 (15)	1.058 (17)	1.025 (16)
	1.06	1.013 (8)	1.018 (8)	1.035 (7)	1.002 (7)	1.003 (9)	1.035 (8)	1.008 (7)
	1.55	1.001 (3)	1.002 (3)	1.015 (3)	1.000 (3)	1.004 (8)	1.013 (3)	1.005 (3)
10 MV FFF	0.58	1.026 (25)	1.082 (22)	1.051 (23)	1.070 (29)	1.072 (26)	1.129 (26)	1.039 (22)
	0.86	1.018 (10)	1.029 (9)	1.024 (10)	1.011 (10)	1.029 (11)	1.048 (14)	1.026 (11)
	1.04	1.020 (6)	1.017 (6)	1.018 (9)	1.003 (6)	1.023 (12)	1.033 (14)	1.015 (6)
	1.52	1.002 (3)	1.007 (3)	1.011 (4)	1.001 (3)	1.006 (3)	1.013 (3)	1.009 (3)
Varian TrueBeam								
6 MV WFF	0.56	1.041 (34)	1.102 (30)	1.085 (32)	1.029 (38)	1.053 (30)	1.181 (34)	1.065 (30)
	0.81	1.012 (10)	1.024 (10)	1.041 (11)	0.998 (11)	1.021 (10)	1.063 (11)	1.020 (10)
	1.01	1.003 (6)	1.008 (5)	1.025 (6)	0.997 (6)	1.011 (6)	1.031 (6)	1.009 (5)
	1.50	0.999 (2)	0.996 (2)	1.009 (2)	0.999 (2)	1.007 (3)	1.008 (2)	1.000 (2)
6 MV FFF	0.54	1.042 (30)	1.101 (26)	1.082 (28)	1.036 (33)	1.047 (26)	1.180 (30)	1.063 (24)
	0.82	1.010 (9)	1.023 (9)	1.037 (9)	0.999 (10)	1.006 (10)	1.047 (9)	1.020 (8)
	0.99	1.002 (5)	1.009 (5)	1.021 (5)	0.998 (6)	1.004 (6)	1.022 (5)	1.008 (5)
	1.49	0.998 (2)	0.998 (3)	1.008 (2)	1.002 (2)	1.001 (2)	1.006 (2)	1.000 (2)
10 MV WFF	0.5	1.040 (32)	1.105 (28)	1.095 (31)	1.033 (35)	1.066 (28)	1.177 (32)	1.068 (27)
	0.8	1.014 (12)	1.032 (11)	1.051 (12)	0.998 (12)	1.028 (12)	1.067 (12)	1.025 (11)
	1.0	1.007 (7)	1.016 (7)	1.034 (7)	0.997 (7)	1.017 (7)	1.039 (7)	1.013 (7)
	1.5	1.000 (3)	1.000 (3)	1.015 (3)	0.998 (3)	1.006 (3)	1.014 (3)	1.006 (3)
10 MV FFF	0.55	1.042 (28)	1.100 (25)	1.088 (27)	1.038 (31)	1.060 (25)	1.183 (28)	1.064 (26)
	0.81	1.015 (10)	1.029 (10)	1.047 (10)	1.000 (11)	1.021 (10)	1.060 (10)	1.022 (10)
	1.02	1.007 (6)	1.015 (6)	1.030 (6)	0.999 (6)	1.012 (6)	1.033 (6)	1.011 (6)
	1.51	1.001 (3)	1.000 (2)	1.013 (3)	0.999 (3)	1.006 (3)	1.013 (3)	1.002 (3)

Detailed analysis show that for the IBA CC04 ionization chamber, statistically significant differences in $k_{Q_{\mathrm{clin}},Q_{\mathrm{ref}}}^{f_{\mathrm{clin}},f_{\mathrm{ref}}}$ were found with respect to the orientation (perpendicular vs parallel: see Fig. 1) of the chambers in all beams for two smallest fields 0.5 cm^2^ × 0.5 cm^2^ and 0.8 cm^2^ × 0.8 cm^2^. Such a finding is to some extent surprising, since the cavity dimensions of the IBA CC04 chamber are comparable along the two main axes — the diameter of the cavity is 4.0 mm, while the length of the cavity is 3.6 mm (Table 1). This results in a very similar volume averaging effect for both perpendicular and parallel orientation, which is the main contributing factor to the output correction factors in that case. It has to be noted that output correction factors were also in this case smaller in parallel orientation vs perpendicular for smallest field sizes ranging from 0.5 cm^2^ × 0.5 cm^2^ to 1.0 cm^2^ × 1.0 cm^2^ for all combination of beam energy, filtration, and linac (Tables 2, 3, 4, 5).

For the PTW 31021 Semiflex 3D chamber, we found lowest number of statistically significant differences among all investigated chambers. On the Elekta linac, ratios $k_{\mathrm{perp}}/k_{\mathrm{para}}$ differ significantly from unity for four setups: in 6 and 10 MV WFF beams for smallest field size of 0.5 cm^2^ × 0.5 cm^2^, and for two smallest fields in 10 MV FFF beam. On Varian TrueBeam linac ratios $k_{\mathrm{perp}}/k_{\mathrm{para}}$ differ significantly from unity only for two setups: in 6 and 10 MV FFF beams for the smallest field size 0.5 cm^2^ × 0.5 cm^2^. Geometrical characteristics of the chamber are presumably the reasons for a similar response in both orientations, since the length of the cavity of 4.8 mm is equal to the cavity diameter (Table 1).

Two more ionization chambers used in the present study, PTW 31016 3D PinPoint and its successor PTW 31022 3D PinPoint, are classified as 3D chambers meaning that “relative dose distributions can be measured with high spatial resolution in any direction” (manufacturer’s statement). Latter characteristic also indicates that there should be only small (negligible) difference in their response regardless of the orientation, perpendicular or parallel, in the beam. Both chambers have a cavity length of 2.9 mm, which is identical to their cavity diameters. However, in this case, differences between the two orientations were found to be more prominent than in the case of previous two examples. Namely, for the PTW 31016 chamber, significant differences are observed for all small fields up to the 1.5 cm^2^ × 1.5cm^2^: on both linacs in all beams. For this chamber, equivalency regarding the orientation has not been observed for all four smallest fields, despite their 3D geometry. Similar, but less pronounced differences were seen also for PTW 31022 chamber.

We have also studied the response of two ionization chambers, which have elongated cavities, having a cavity dimension along the principal chamber’s axis (direction of the central electrode) larger than the diameter of the cavity. These two chambers are IBA Razor and PTW 31023 PinPoint. Both chambers have a cavity diameter of 2.0 mm. However, they have different cavity lengths: IBA Razor has a cavity length of 3.6 mm, while the PTW 31023 PinPoint chamber has a cavity of length 5.0 mm (Table 1). For IBA Razor chamber, significant differences were found for the output correction factors only for smallest fields up to 1.0 cm^2^ × 1.0 cm^2^ field size in all photon beams on both linacs, while for the PTW 31023 chamber, significant differences in the response were found for four smallest investigated field sizes (up to 1.5 cm^2^ × 1.5 cm^2^) on both linacs. The most pronounced differences, concerning the orientation, were found for the PTW 31023 PinPoint chamber, which was expected, since this chamber has the most elongated cavity geometry among all chambers included in the study, and thus the most notably expressed volume averaging effect in the perpendicular orientation. For IBA Razor chamber, we noticed a slight overresponse for smallest field sizes in particular in 10 MV FFF beam on Elekta Versa HD (Table 3). The resulting fitting curve lies completely below x‐axis (Fig. 3) because output correction factor is below unity also for the smallest field 0.5 cm^2^ × 0.5 cm^2^, which was not the case for other setups. Only in this case, the fitting parameter c from Eq. (8) was different from 0.

The smallest volume chamber in our study, SI Exradin A16, showed significant differences in $k_{Q_{\mathrm{clin}},Q_{\mathrm{ref}}}^{f_{\mathrm{clin}},f_{\mathrm{ref}}}$ with respect to the orientation in all four smallest fields on Elekta linac and for three smallest fields on Varian linac, regardless of the beam energy and filtration. Exradin A16 chamber has cavity dimensions equal in both chamber’s axis, similar to PTW 31016 and PTW 31022 3D PinPoint chambers discussed earlier, which could qualify also this chamber to the group of “3D chambers.” Its cavity volume of 0.007 cm^3^ is two times smaller than the corresponding volumes of the PTW 31016 and PTW 31022 3D chambers. These geometrical characteristics of the SI Exradin A16 chamber minimize the volume averaging effect in both orientations.

All values of ratios $k_{\mathrm{perp}}/k_{\mathrm{para}}$ (Eq. 12) of differences in $k_{Q_{\mathrm{clin}},Q_{\mathrm{ref}}}^{f_{\mathrm{clin}},f_{\mathrm{ref}}}$ with respect to the orientation in the beam are given in Table 4 for all ionization chambers included in the study and for four smallest field sizes. Associated combined absolute uncertainties $u_{C}$ are presented with a coverage factor k = 2.

Finally, we have compared output correction factors for two ionization chambers in two orientations, PTW 31022 3D PinPoint and PTW 31023 PinPoint, which have very similar cavity volumes but considerably different ratios $d_{L}/d_{R}$ between respective cavity lengths in longitudinal and radial directions. From the plot in Fig. 6, we can see that $k_{\mathrm{perp}}/k_{\mathrm{para}}$ for a particular equivalent square small field size $S_{\mathrm{clin}}$ notably depends on the $d_{L}/d_{R}$ ratio, displaying higher values for more elongated chamber PTW 31023 Pinpoint. In addition, $k_{\mathrm{perp}}/k_{\mathrm{para}}$ values gradually approach to unity for both chambers with increasing field size. For field size 1.5 × 1.5 cm^2^, there is no apparent difference between correction factors determined in both orientations, that is, $k_{\mathrm{perp}}/k_{\mathrm{para}}\approx 1$.

To summarize, output correction factors for all ionization chambers included in the present study are lower if the chambers are oriented with their main axis parallel to the central axis of the beam even if the length of the cavity is equal to the cavity diameter as it is in the case of 3D ionization chambers. Consequently, from the point of view of minimizing values of output correction factors $k_{Q_{\mathrm{clin}},Q_{\mathrm{ref}}}^{f_{\mathrm{clin}},f_{\mathrm{ref}}}$, the use of ionization chambers in parallel orientation is advantageous compared to perpendicular orientation for the determination of field output factors in small static fields in megavoltage photon beams. However, until independent validation of our results and possible update of TRS‐483 CoP, clinical users are advised to follow the recommendations given in TRS‐483.

We recommend that the guidance given in IAEA TRS‐483, that $0.95<k_{Q_{\mathrm{clin}},Q_{\mathrm{ref}}}^{f_{\mathrm{clin}},f_{\mathrm{ref}}}<1.05,$ continued to be followed for the selection of beam energy/field size/ionization chambers for measurements of field output factors in small static fields in high energy photon beams regardless of the orientation of the chamber long axis with respect to the beam central axis. Five ionization chambers from our study set fulfill this requirement down to the field size of 0.8 cm^2^ × 0.8 cm^2^ for every investigated megavoltage beam on both linacs if they are used in parallel orientation: IBA Razor, PTW 31016 3D PinPoint, PTW 31022 3D PinPoint, PTW 31023 PinPoint, and SI Exradin A16. It should be noted that two of these five chambers, IBA Razor IC and PTW 31023 PinPoint, comply with the requirement $0.95<k_{Q_{\mathrm{clin}},Q_{\mathrm{ref}}}^{f_{\mathrm{clin}},f_{\mathrm{ref}}}<1.05$ even down to the smallest investigated field size of 0.5 cm^2^ × 0.5 cm^2^ when they are positioned in the parallel orientation.

Lastly, while the output correction factors determined in our study represent “total” output correction factors, and thus include potential variations of the polarity correction and ion recombination factor in small fields, it is still recommended, to investigate the size of that effect, before using ionization chambers for measurements in small photon fields.

## 5 Summary

The present paper provides experimental results of detector‐specific output correction factors $k_{Q_{\mathrm{clin}},Q_{\mathrm{ref}}}^{f_{\mathrm{clin}},f_{\mathrm{ref}}}$ for seven small volume ionization chambers, determined in perpendicular and parallel orientation with respect to the central axis of the beam (Fig. 1). Reference values for field output factors were obtained with two detectors, plastic scintillator Exradin W1 and EBT3 radiochromic film, following the methodology developed by our group and thoroughly described earlier.^32^ All measurements were performed at Elekta Versa HD and Varian TrueBeam linear accelerators in 6 and 10 MV photon beams with and without flattening filter, down to the smallest field size of 0.5 cm^2^ × 0.5 cm^2^.

This large set of output correction factors for seven ionization chambers determined in perpendicular orientation is considered a valuable supplement to the TRS‐483 dataset, in particular for four ionization chambers IBA Razor IC, PTW 31021 3D Semiflex, PTW 31022 PinPoint 3D, and PTW 31023 PinPoint for which TRS‐483 did not provide $k_{Q_{\mathrm{clin}},Q_{\mathrm{ref}}}^{f_{\mathrm{clin}},f_{\mathrm{ref}}}$ values. Detector‐specific output correction factors obtained in our study for the remaining three ionization chambers (IBA CC04, PTW 31016 PinPoint 3D, and SI Exradin A16) in perpendicular orientation, confirm the corresponding data published in the TRS‐483 using the 95% confidence limits.

In addition to the output correction factors determined in perpendicular orientation, we have also provided a large set of $k_{Q_{\mathrm{clin}},Q_{\mathrm{ref}}}^{f_{\mathrm{clin}},f_{\mathrm{ref}}}$ values for the same set of small volume ionization chambers in parallel orientation [Fig. 1(b)]. Our main finding is that output correction factors were lower if they were determined in a parallel orientation of ionization chambers compared to those obtained in a perpendicular orientation for all seven ionization chambers regardless of the photon beam energy, filtration, or linear accelerator being used. Consequently, if the parallel orientation is utilized for the determination of output correction factors, they can be determined for field sizes smaller than those reported in the TRS‐483, since the requirement $0.95<k_{Q_{\mathrm{clin}},Q_{\mathrm{ref}}}^{f_{\mathrm{clin}},f_{\mathrm{ref}}}<1.05$ is not violated even for field sizes below 1.0 cm^2^ × 1.0 cm^2^ for five ionization chambers used in the present study. Additionally, output correction factors $k_{Q_{\mathrm{clin}},Q_{\mathrm{ref}}}^{f_{\mathrm{clin}},f_{\mathrm{ref}}}$ determined with IBA Razor and PTW 31023 PinPoint ionization chambers in a parallel orientation were always within the interval 0.95–1.05 regardless of the photon beam energy or linac used down to the smallest investigated field size of 0.5 cm^2^ × 0.5 cm^2^.

To conclude, for minimizing corrections in the experimental determination of field output factors, parallel orientation of ionization chambers is advantageous over the perpendicular. This latter outcome of our study is considered as a valuable contribution to the discussion on the orientation of ionization chambers in small MV beams. While the results from the present study justify our final statement, further investigations and confirmation of our findings regarding the orientation of ionization chambers in small fields, from other research groups, are necessary for an eventual update of the TRS‐483 in the future. Until that happens, the recommendations from TRS‐483 should be followed.
